# An intensive neurofeedback alpha-training to improve sleep quality and stress modulation in health-care workers during the COVID-19 pandemic: A pilot study

**DOI:** 10.1192/j.eurpsy.2021.705

**Published:** 2021-08-13

**Authors:** D. Conti, L. Celebre, N. Girone, L. Molteni, M. Vismara, B. Benatti, M. Bosi, A. Colombo, B. Dell’Osso

**Affiliations:** Luigi Sacco University Hospital, Psychiatry 2 Unit., University of Milan, Italy., Milan, Italy

**Keywords:** sleepquality, Neurofeedback, stress, COVID-19

## Abstract

**Introduction:**

During the COVID-19 pandemic, health workers represented a group particularly vulnerable to work-related stress, but prevention and management of psychiatric symptoms are still under evaluation. Neurofeedback is a safe and non-invasive neuromodulation technique with the target of training participants in the self-regulation of neural substrates underlying specific psychiatric disorders. Protocols based on the increase of alpha frequencies, associated with the process of relaxation, are used for the treatment of stress, anxiety and sleep disturbances.

**Objectives:**

The aim of the present study was to assess the effectiveness of an alpha-increase NF protocol for the treatment of stress in healthcare workers exposed to the COVID-19 pandemic.

**Methods:**

Eighteen medical doctors belonging to the Sacco Hospital were recruited during the COVID-19 health emergency and underwent a 10 sessions NF alpha-increase protocol during two consecutive weeks. The level of stress was assessed at the beginning (T0) and at the end (T1) of the protocol through the following questionnaires: Severity of Acute Symptoms Stress (SASS), Copenhagen Burnout Inventory (CBI), Pittsburgh Sleep Quality Index (PSQI), Brief-COPE. Statistical analyses were performed with Paired Samples t-Test for continuous variables, setting significance at p < 0.05.

**Results:**

A significant increase in alpha waves mean values between T0 and T1 was observed. In addition, a significant reduction in the PSQI test score between T0 and T1 was observed.

**Conclusions:**

Alpha-increase protocol showed promising results in terms of stress modulation, sleep quality improvement and safety profile in a pilot sample of health-care workers. Larger controlled studies are warranted to confirm present results.

